# Effects of Hydrogenation on the Corrosion Behavior of Zircaloy-4

**DOI:** 10.3390/ma17051101

**Published:** 2024-02-28

**Authors:** Huifang Yue, Mingyang Zhou, Yanli Zhao, Yinjie Han, Shichao Liu, Laiyao Geng, Shitong Xu, Yong Xin, Meiyi Yao

**Affiliations:** 1 Science and Technology on Reactor System Design Technology Laboratory, Nuclear Power Institute of China, Chengdu 610213, China; npic201706@163.com (H.Y.); 18782951516@163.com (M.Z.);; 2 Institute of Materials, Shanghai University, Shanghai 200072, China

**Keywords:** fuel cladding, pre-hydrogenation, corrosion kinetics, Raman spectroscopy, zirconium hydride

## Abstract

Hydrogen plays an important role in the corrosion of zirconium alloys, and the degree of influence highly depends on the alloy composition and conditions. In this work, the effects of hydrogenation on the corrosion behavior of Zircaloy-4 in water containing 3.5 ppm Li + 1000 ppm B at 360 °C/18.6 MPa were investigated. The results revealed that hydrogenation can shorten the corrosion transition time and increase the corrosion rates of Zircaloy-4. The higher corrosion rates can be ascribed to the larger stress in the oxide film of hydrogenated samples, which can accelerate the evolution of the microstructure of the oxide film. In addition, we also found that hydrogenation has little effect on the t-ZrO_2_ content in the oxide film and there is no direct correspondence between the t-ZrO_2_ content and the corrosion resistance of the Zircaloy-4.

## 1. Introduction

As the primary barrier for containing radioactive fission products, the performance of fuel cladding directly impacts the safety and reliability of nuclear reactors. Zirconium alloys are a preferred material for commercial pressurized water reactor fuel cladding due to their exceptional corrosion resistance, mechanical properties, and thermal neutron economy. However, zirconium alloys readily absorb hydrogen through oxidation reactions with cooling water [1]. The solubility of hydrogen in zirconium alloys is relatively low, approximately 80 ppm at 300 °C and 200 ppm at 400 °C. Consequently, exceeding the solubility limit leads to the precipitation of brittle zirconium hydride [2]. The formation of zirconium hydride not only diminishes the mechanical properties but also influences the corrosion behavior of zirconium alloys [3,4,5,6].

The corrosion behavior of different zirconium alloys in various environments under the influence of hydrogen has been extensively investigated. However, due to its complex nature, the research on its mechanism is still incomplete and the results remain controversial. Ensor et al. [7] examined the impact of hydrogen on the corrosion behavior of Zircaloy-4 in 360 °C water and observed that higher hydrogen content beyond terminal solid solubility resulted in increased corrosion rates. This was attributed to the formation of precipitated hydrides, which increased the surface area between the metal and oxide layer, thereby facilitating greater access for oxidizing species to react with the metal. Kim et al. [8] investigated how hydrogen affects the corrosion of Zircaloy-4 and Zr-1.5Nb alloy in 600 °C air and found that pre-hydrided alloys consistently exhibited lower fractions of the t-ZrO_2_ phase compared to un-hydrided ones. Arashi et al. [9] demonstrated that the stability of the t-ZrO_2_ phase is dependent on stress conditions; thus, precipitation of hydrides reduced the Pilling–Bedworth ratio from 1.56 to 1.29, relieving compressive stress at interfaces and promoting transformation from protective t-ZrO_2_ to the non-protective m-ZrO_2_ phase, consequently accelerating the corrosion rate. The importance of the Pilling–Bedworth ratio as a criterion for assessing the integrity of oxide films has also been highlighted by Wu et al. [10], while the PB ratio is intricately linked to the phase composition fraction in the oxide film. However, Mao et al. [11] investigated the corrosion resistance of six zirconium alloy samples, including Zr-4, under superheated steam conditions at 400 °C and 10.3 MPa pressure. Their findings suggest that hydrogen has a negligible impact on the corrosion resistance exhibited by these zirconium alloys. Similarly, Yao et al. [12] and Chu et al. [13] have also reported that there is no strict correlation between the degree of hydrogen absorption in zirconium alloys and their level of corrosion resistance. Furthermore, Kim et al. [14] studied the corrosion behavior of pre-hydrogenated Zircaloy-4 alloy in a 350 °C LiOH aqueous solution and discovered that specimens charged with hydrogen exhibited accelerated corrosion due to the rapid oxidation of Zr-hydride within a short period; however, slower corrosion rates were observed over longer periods due to the presence of pre-existing Zr-hydride inhibiting further uptake into the specimen. Moreover, the research conducted by Xu et al. [15] demonstrated that appropriate hydrogen can improve the corrosion resistance of zirconium alloy in a 360 °C LiOH aqueous solution.

The influence of hydrogen on the corrosion behavior of zirconium alloys is closely associated with the specific corrosion conditions. Most existing studies have focused on investigating the impact of hydrogen on zirconium corrosion in pure water, lithium aqueous solutions, or high-temperature air. Despite significant advancements achieved in these investigations, the research above indicates that the underlying mechanism remains unclear and lacks consistency. Therefore, further research is still needed.

This study investigates the impact of hydrogenation on the corrosion behavior of Zircaloy-4 in high-temperature and high-pressure water containing Li and B, simulating the typical cooling water environment of commercial pressurized water reactors. It can provide valuable technical guidance and theoretical support for enhancing the performance of Zircaloy-4 as fuel cladding.

## 2. Experimental Procedure

### 2.1. Materials and Preparation

A commercial recrystallized annealed Zircaloy-4 (Zr-1.35Sn-0.21Fe-0.11Cr, wt%; the Zr content is approximately 98.33 wt%) alloy plate with a thickness of 0.7 mm was selected and cut into corrosion samples measuring 20 mm × 15 mm × 0.7 mm in dimensions. The Zircaloy-4 plate was obtained from a fuel cladding supplier and subjected to recrystallization annealing in a vacuum furnace (DL2890 horizontal vacuum annealing furnace, Lanzhou Vacuum Equipment Limited Liability Company, Lanzhou, China). Each sample underwent drilling, grinding, and cleaning using a mixed-acid solution (30% H_2_O + 30% HNO_3_ + 30% H_2_SO_4_ + 10% HF by volume) followed by rinsing with deionized water. Subsequently, the gas-phase hydrogenation method was employed for pretreating the samples in a tube furnace (Lindberg/Blue M, Shanghai Danding International Trade Limited Liability Company, Shanghai, China), where the hydrogenation temperature was set at 400 °C while using a gas mixture of 4% H_2_ + 96% Ar. After hydrogenation, the samples were homogenized at 400 °C in a vacuum annealing furnace (UnitemP RTP-150, UniTemp GmbH, Pfaffenhofen, Germany). Three levels of hydrogen content were established for each alloy: no hydrogenation treatment; approximately 240 ppm (twice the solid solubility of hydrogen in α-Zr at a corrosion temperature of 360 °C); and approximately 360 ppm (three times the solid solubility of hydrogen in α-Zr at 360 °C). All samples were scrubbed with an etching solution consisting of 10% H_2_O_2_ + 80% HNO_3_ + 10% HF before being observed under an optical microscope. The O-N-H analyzer (Leco-RH 600, LECO, Saint Joseph, MO, USA) was used to determine their respective hydrogen contents. If the desired value was not achieved, the aforementioned steps would be repeated until it met the requirements. Once reaching the target value, the samples would undergo pre-treatment, followed by acid washing, rinsing in deionized water, drying, and weighing prior to corrosion testing.

### 2.2. Material Characterization

The corrosion test was conducted at 360 °C/18.6 MPa in water containing 3.5 ppm Li+ and 1000 ppm B. Deionized water with a resistivity exceeding 1 MΩ was employed as the corrosive medium for the experiment. The exposure durations were set as follows: 3 d, 14 d, 42 d, 70 d, 100 d, 130 d, 160 d, and 190 d for weight gain assessment. Subsequently, the weight gains per unit surface area (∆*W*) in mg/dm^2^ were determined using the following equation:

(1) $$\Delta W=\frac{W_{1}-W_{2}}{S}$$

where *W*_1_ and *W*_2_ represent the specimen weight after corrosion and before corrosion, respectively. *S* represents the specimen surface area.

Three specimens for each exposure time were tested. The corrosion resistance of the materials was evaluated by calculating the average weight gain of three parallel specimens for each exposure time.

The microstructure of all samples was observed before and after the corrosion test using an OLYMPUS-GX53 (Olympus Corporation, Tokyo, Japan) inverted metallurgical microscope to evaluate the morphology of zirconium hydride, grain morphology, and grain size. A high-resolution scanning electron microscope (HRSEM, JSM-7500F, JEOL, Tokyo, Japan) was used to observe the morphology and size of the second phase in the alloy. The microstructure and micro-zone composition of the alloy were analyzed using a high-resolution transmission electron microscope (HRTEM, JEM-200CX, JBOL, Tokyo, Japan) equipped with an energy spectrometer (EDS), while crystal structure analysis of the second phase was conducted by photographing selected area electron diffraction (SAED) patterns or high-resolution lattice streak images (HRTEM images). Additionally, SEM was utilized to observe oxide film fracture morphology on corroded samples as well as measure oxide film thickness. Microstructure observation and analysis for oxide film cross-sections on corrosion samples were performed using a TEM equipped with EDS. Finally, Raman peaks from corroded sample oxide films were obtained through the use of an Invia Qontor confocal micro-Raman spectrometer (Invia Oontor, Renishaw, London, UK), which allowed for the analysis of phase composition via a peak-fitting process through NGS Labspec software (Version: 5.58.25).The t-ZrO_2_ content percentage and isostatic pressure were determined using Equations (2) and (3) [16]:

(2) $$f_{tet}=\frac{I_{t}\left(280\right)}{I_{m}\left(178\right)+I_{m}\left(192\right)+I_{t}\left(280\right)}$$

(3) Δv=A · ΔP

where *I_m_* and *I_t_* represent the intensities of Raman peaks corresponding to m-ZrO_2_ and t-ZrO_2_, respectively. In Δ*ν* = *ν* − *ν*_0_, Δ*ν* represents the drift size of the Raman peak position, *ν* represents the Raman peak position in the presence of the internal stress in the oxide film, and *ν*_0_ denotes the Raman peaks of the ZrO_2_ powders without stress. Δ*P* represents the isostatic pressure.

## 3. Results and Discussion

### 3.1. Microstructure of Zircaloy-4 before and after Hydrogenation

**Figure 1:**
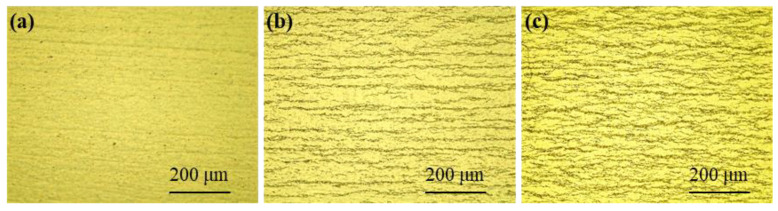
Metallographic image of Zircaloy-4 samples: (**a**) without hydrogenation, (**b**) low-level hydrogen, and (**c**) high-level hydrogen.

**Figure 2:**
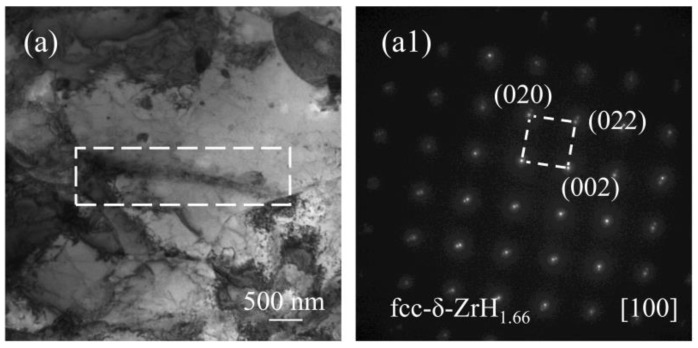
TEM bright-field image (**a**) and corresponding SAED pattern (**a1**) of high-hydrogen sample.

Metallographic images of zirconium hydride in Zircaloy-4 before and after hydrogenation are presented in Figure 1. It can be observed that the non-hydrotreated sample exhibits minimal detection of zirconium hydride, indicating a low hydrogen content. Conversely, the hydrogenated samples display elongated strip-like hydrides that are predominantly parallel to each other [17,18,19], with a uniform distribution throughout the alloy. For further analysis of the morphology and crystal structure of hydrogenated zirconium, Figure 2 illustrates results obtained from a high-hydrogen sample. By combining Figure 1 and Figure 2, it becomes evident that upon hydrogenation, the concentration of hydrogen in Zircaloy-4 samples surpasses their solid solubility threshold, resulting in the formation of δ-ZrH_1.66_ with a face-centered cubic (fcc) structure as determined through calibration and analysis. Previous studies [20] have indicated four types of precipitated hydrides in zirconium alloys: ζ, γ, δ, and ε phases. Among these phases, ζ and γ are considered unstable while the ε phase only exists at very high levels of hydrogen content. Due to post-hydrogenation stabilization treatment applied to our samples, stable δ-phase ZrH_1.66_ was achieved, this being the most prevalent form of hydride found within zirconium alloys [21,22].

### 3.2. Effect of Hydrogenation on the Corrosion Kinetics of Zircaloy-4

**Figure 3:**
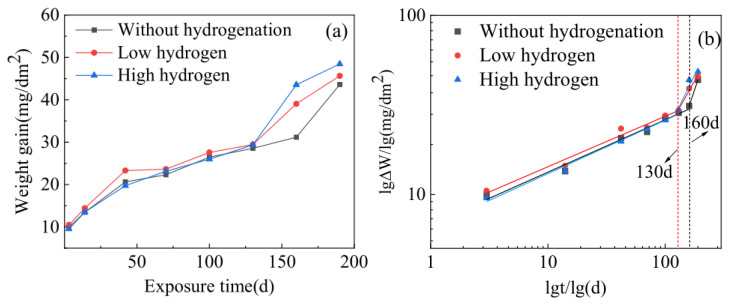
Corrosion weight gain curves of three samples of Zircaloy-4 alloy with different hydrogen contents in aqueous solution of 360 °C/18.6 MPa/3.5 ppm Li + 1000 ppm B: (**a**) natural number coordinates, (**b**) double logarithmic coordinates.

The corrosion weight gain curves of Zircaloy-4 samples with different hydrogen contents in a 360 °C/18.6 MPa/3.5 ppm Li + 1000 ppm B aqueous solution are presented in Figure 3. The weight gain shown in Figure 3 represents the average value of three parallel specimens for each case, and the deviation among the results of these specimens is negligible. As depicted in Figure 3, all three types of samples exhibited distinct corrosion transitions. For the non-hydrogenated samples, this transition occurred at 160 days; however, both low-hydrogen and high-hydrogen samples experienced it at 130 days instead. The growth kinetics of zirconium alloy oxide film can be divided into two stages: before the transition, the corrosion rate is low and follows an approximately cubic weight gain curve; after the transition, there is an increase in corrosion rate and a shift towards an approximately linear weight gain curve [23]. It was observed that hydrogenation advanced the transition time for these samples, indicating that it accelerates Zircaloy-4’s corrosion process. Furthermore, when exposed for a duration of 190 days, high-hydrogen and low-hydrogen Zircaloy-4 samples exhibited corrosion weight gains of 48.6 mg/dm^2^ and 45.7 mg/dm^2^, respectively, while non-hydrogenated samples showed a corrosion weight gain of only 43.6 mg/dm^2^. This suggests that increasing hydrogen content to as much as 360 ppm reduces Zircaloy-4’s corrosion resistance by 11.5%.

### 3.3. Surface and Fracture Morphology of the Oxide Film

**Figure 4:**
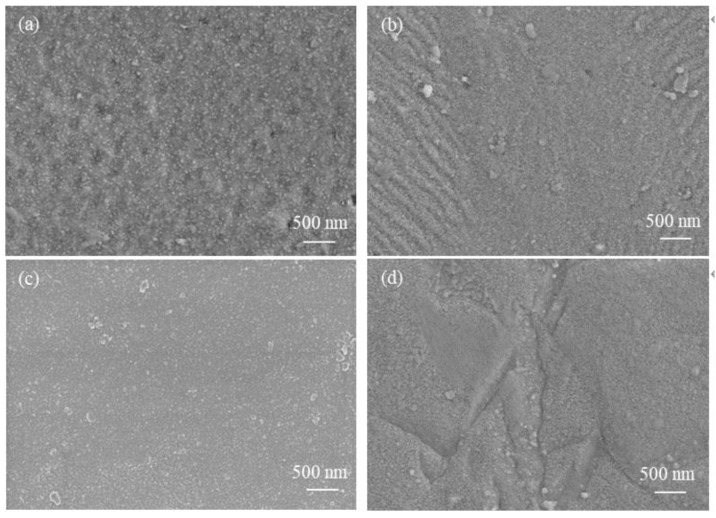
SEM images of oxide film surfaces of non-hydrogenated (**a**,**b**) and high-hydrogen (**c**,**d**) samples of Zircaloy-4 corroded for 70 d (**a**,**c**) and 190 d (**b**,**d**) in aqueous solution of Li + 1000 ppm B at 360 °C/18.6 MPa/3.5 ppm Li + 1000 ppm B.

Figure 4 shows the SEM images of the external surface of the oxide film on non-hydrogenated Zircaloy-4 and on high-hydrogen content Zircaloy-4 after exposure to 360 °C/18.6 MPa/3.5 ppm Li + 1000 ppm B aqueous solution for 70 d and 190 d. Notably, there is minimal disparity observed between the two outer surfaces across both exposure durations. Specifically, after a 70-day exposure period, the outer surface of the oxide film on the two samples appears relatively flat with no discernible cracks present. Similarly, following a prolonged exposure duration of 190 days, most areas on both samples exhibit smooth surfaces; however, parallel rumble strips are evident on the non-hydrogenated sample’s surface due to grain orientation [24].

**Figure 5:**
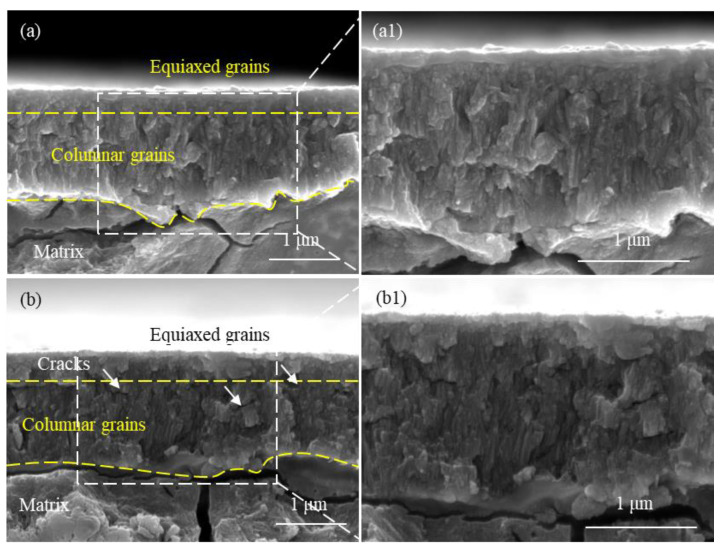
SEM images of oxide film fracture of non-hydrogenated (**a**,**a1**) and high-hydrogen (**b**,**b1**) samples of Zircaloy-4 corroded for 70 d in aqueous solution of 3.5 ppm Li + 1000 ppm B at 360 °C/18.6 MPa.

**Figure 6:**
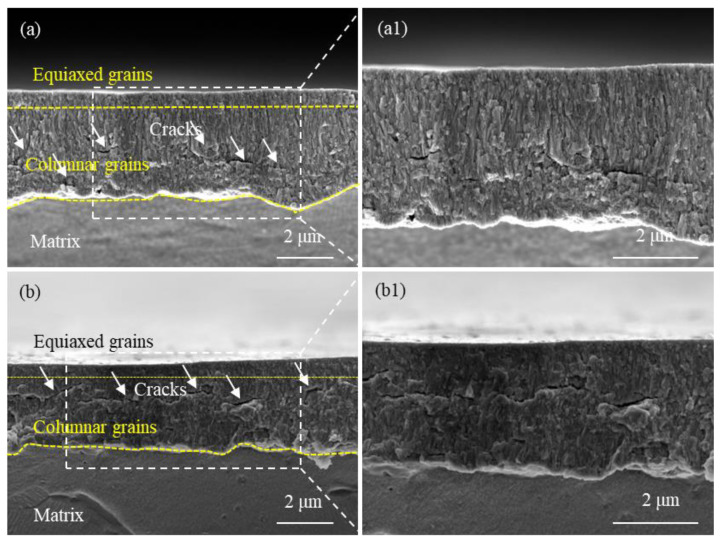
SEM images of oxide film fracture of non-hydrogenated (**a**,**a1**) and high-hydrogen (**b**,**b1**) samples of Zircaloy-4 corroded for 190 d in aqueous solution of 3.5 ppm Li + 1000 ppm B at 360 °C/18.6 MPa.

The SEM images in Figure 5 and Figure 6 depict the cross-sections of a non-hydrogenated sample and a high-hydrogen sample after exposure to a 360 °C/18.6 MPa/3.5 ppm Li + 1000 ppm B aqueous solution for 70 d and 190 d, respectively. Following a corrosion period of 70 days, both the non-hydrogenated sample and high-hydrogen sample exhibited oxide films predominantly composed of columnar crystals, with a few equiaxial crystals observed near the outer region of the film. The oxide film on the non-hydrogenated sample appeared intact and dense without any noticeable microcracks (Figure 5a,a1). However, there were a few microcracks present in the oxide film on the high-hydrogen sample (Figure 5b,b1). After an extended corrosion time of 190 days, both samples displayed oxide films consisting mainly of columnar crystals along with equiaxial crystals. Additionally, cracks parallel to the O/M interface were observed in both samples’ oxide films; however, these cracks became more numerous and larger in size within the high-hydrogen sample compared to those found in the non-hydrogenated one (Figure 6a,a1,b,b1). The cracks were transverse and located closer to the outside of the oxide film.

According to the studies conducted by Bai et al. and Wang et al. [25,26], prior to the corrosion transition, the oxide film primarily consists of columnar crystals with minimal presence of pores and cracks. The corrosion transition occurred at 160 days for the non-hydrogenated sample and at 130 days for the high-hydrogen sample. After 70 days of corrosion, no corrosion transition was observed in either type of sample, and both exhibited an oxide film dominated by columnar crystals. Yilmazbayhan et al. [27] reported that columnar grains provide better protection against further oxidation due to their significantly higher diffusion coefficient compared to that of oxide. The reduction in grain boundary area resulting from larger columnar grains limits oxygen diffusion paths, leading to lower matrix corrosion and fewer cracks in the oxide film after 70 days of corrosion. As the duration of corrosion increases, there is a gradual transformation from columnar grains to equiaxial grains in the outer layer of the oxide film, resulting in increased grain boundary density and oxygen diffusion channels, consequently accelerating the corrosion rate [28]. When both non-hydrogenated and high-hydrogen samples were corroded for 190 days, more equiaxial grains appeared, which accelerated corrosion progression in both cases. The P.B. ratio of Zr oxidized to ZrO_2_ is 1.56 with volume expansion occurring during the oxidation process. Due to constraints imposed by the metal matrix, internal compressive stress develops within the oxide film, leading to numerous cracks [29]. With longer durations of corrosion time, thicker oxide films are formed along with greater internal stress within them; hence, more cracks appeared on both samples after being corroded for 190 days.

In this study, after 190 days of corrosion, significant large cracks were observed in the oxide film of the high-hydrogen sample (Figure 6b,b1). This occurrence can be attributed to the increased hydrogen production from pre-formed zirconium hydride during oxidation. According to Kuwae’s hydrogen aggregation model [30], hydrogen tends to aggregate during Zr oxidation. When the pressure exceeds the compressive limit of the oxide film, it leads to its rupture and consequently accelerates alloy corrosion. Therefore, at a corrosion time of 190 d, there are more and larger cracks present in the outer region of the oxide film on high-hydrogen samples compared to non-hydrogenated samples. These cracks serve as channels for O^2−^ or OH^−^ diffusion [31], which further accelerates zirconium alloy corrosion. This could potentially explain why high-hydrogen samples exhibit an advanced corrosion transition time.

### 3.4. TEM Analysis of the Microstructure of the Cross-Section of the Oxide Film

**Figure 7:**
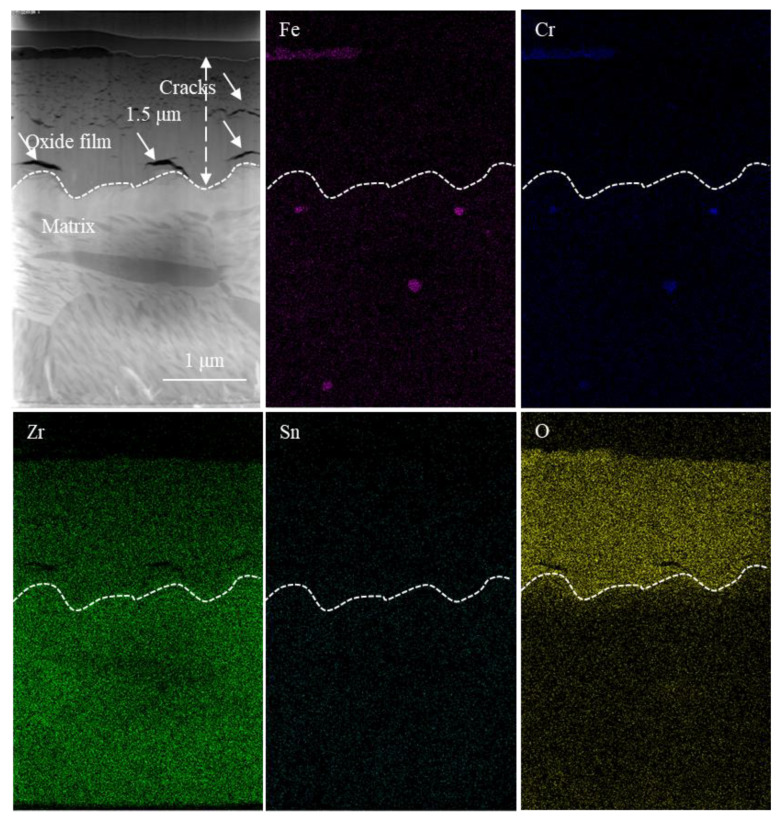
HAADF image of the cross-section microstructure of the oxide film and EDS surface distribution of corresponding elements (Fe, Cr, Zr, Sn, O) of the non-hydrogenated sample of Zircaloy-4 exposed to 360 °C/18.6 MPa/3.5 ppm Li + 1000 ppm B aqueous solution for 70 d.

**Figure 8:**
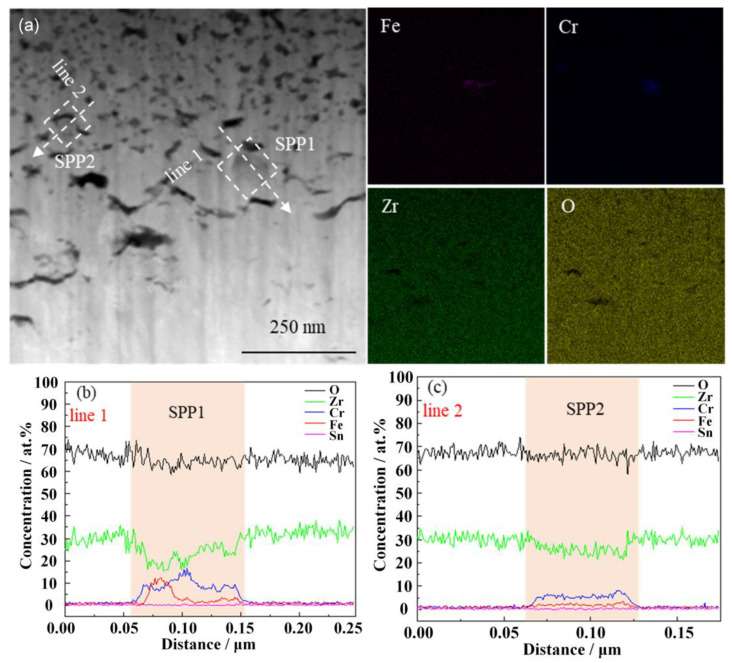
HAADF image of the localized oxide film cross-section microstructure of the non-hydrogenated sample of Zircaloy-4 exposed to 360 °C/18.6 MPa/3.5 ppm Li + 1000 ppm B aqueous solution for 70 d (**a**) and the EDS surface distributions of the corresponding elements (Fe, Cr, Zr, O) as well as the results of the line scans of SPP1 and SPP2 (**b**,**c**).

The HAADF image (high-angle annular dark field image) of the cross-section microstructure of the oxide film and the EDS surface distribution of the corresponding elements of the non-hydrogenated sample corroded for 70 d are presented in Figure 7. Additionally, Figure 8 displays the HAADF image of the cross-section microstructure of the localized oxide film, along with the EDS surface distribution of the corresponding elements and results from line scans conducted on SPP1 and SPP2. From Figure 7, it is evident that the interface between the oxide and metal (O/M) exhibits undulations, with transverse cracks commonly observed near the metal bulges of this interface, consistent with findings by Weekes et al. [32]. The thickness of the oxide film measures approximately 1.5 μm, and micropores are densely distributed from the middle to outer regions of the film. This distribution may be attributed to stress release during the transformation from columnar grains to equiaxed grains [33]. In Figure 8, a higher number of microcracks can be observed surrounding the second phases of SPP1 and SPP2, which primarily consist of Zr, Fe, Cr, and O elements (Figure 8b,c). Furthermore, both second-phase SPP1 and SPP2 exhibit comparable oxygen content to that found in adjacent regions of the oxide film, indicating oxidation has occurred within these second phases. However, variations in Fe and Cr content suggest diffusion processes involving these elements. Zircaloy-4 exclusively presents a unique type of second phase known as Zr(Fe,Cr)_2_ [34]; thus, prior to oxidation, these phases SPP1 and SPP2 corresponded to Zr(Fe,Cr)_2_. Microcracks were observed around the SPP1 and SPP2 because the second phases near the cracks are more easily oxidized and on the contrary, the second phases subsequently promote crack formation [35]. In addition, during corrosion, Fe diffuses out of the Zr(Fe,Cr)_2_ and is oxidized, which is consistent with previous research [36].

**Figure 9:**
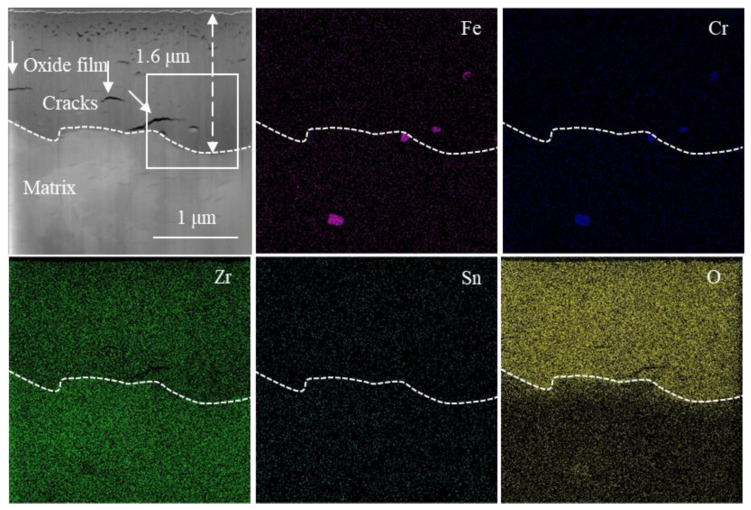
HAADF image of the cross-section microstructure of the oxide film and the EDS surface distribution of the corresponding elements (Fe, Cr, Zr, Sn, O) of a high-hydrogen sample of Zircaloy-4 exposed to 360 °C/18.6 Mpa/3.5 ppm Li + 1000 ppm B aqueous solution for 70 d.

**Figure 10:**
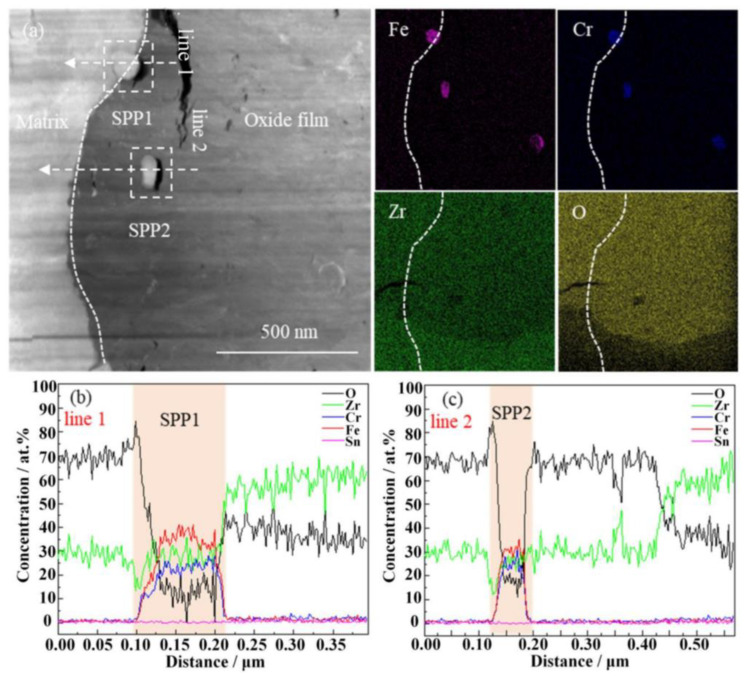
HAADF image of the cross-section microstructure of partial oxide film of a high-hydrogen sample of Zircaloy-4 exposed to 360 °C/18.6 Mpa/Li + B aqueous solution for 70 d (**a**) and EDS surface distribution of the corresponding elements (Fe, Cr, Zr, O) and line scan results of SPP1, SPP2 (**b**,**c**).

The HAADF image in Figure 9 displays the cross-section microstructure of the oxide film, while the corresponding EDS surface distribution reveals the elements present in the Zircaloy-4 high-hydrogen sample after a 70-day exposure. Similarly, Figure 10 presents the HAADF image of the localized oxide film’s cross-section microstructure and its corresponding EDS surface distribution for high-hydrogen samples corroded for 70 days, along with line scan results for SPP1 and SPP2. From Figure 9, it can be observed that the average thickness of the oxide film is approximately 1.6 μm, with visible cracks present within it. Comparatively, when compared to non-hydrogenated samples, both thicker oxide films and larger crack sizes are evident. In Figure 10b,c, O content in the second-phase SPP1 and SPP2 is very low, and the diffusion of Fe and Cr elements basically did not occur. It is evident that Zr(Fe,Cr)_2_ in the high-hydrogen sample remains unoxidized. Pêcheur [37] proposed that the corrosion rate of the second-phase Zr(Fe,Cr)_2_ is comparatively lower than that of the Zr matrix, and after the oxidation of the matrix, the second phase is gradually oxidized. Previous studies have reported a high affinity of the second-phase Zr(Fe,Cr)_2_ for hydrogen absorption [38]. The nearby zirconium hydride provides hydrogen after oxidation for Zr(Fe,Cr)_2_, while unoxidized Zr(Fe,Cr)_2_ in the oxide film acts as a preferential pathway for hydrogen absorption, leading to precipitation of new zirconium hydride. Consequently, this interaction between zirconium hydride and the second phase ultimately accelerates corrosion, resulting in thicker oxide films and larger cracks in high-hydrogen samples compared to non-hydrogenated samples.

### 3.5. Raman Spectroscopy Analysis of the Oxide Film

**Figure 11:**
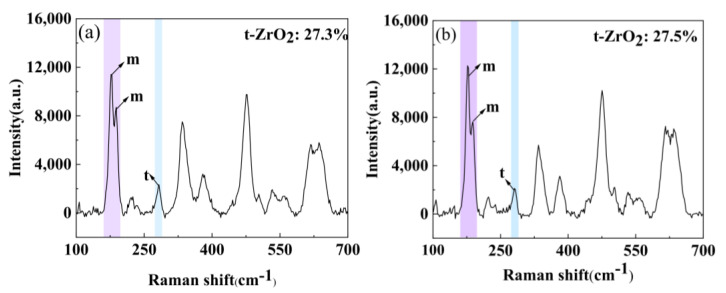
Raman spectroscopy of non-hydrogenated (**a**) and high-hydrogen (**b**) samples of Zircaloy-4 corroded for 70 d in aqueous solution of Li + 1000 ppm B at 360 °C/18.6 MPa/3.5 ppm Li + 1000 ppm B.

The Raman spectral analysis of non-hydrogenated samples and high-hydrogen samples corroded for 70 days is presented in Figure 11 and Table 1. The percentage of the t-ZrO_2_ phase in the oxide film is approximately 27.3% for the non-hydrogenated sample and 27.5% for the high-hydrogen sample, indicating no significant difference between the two samples regarding t-ZrO_2_ phase content in the oxide film. Some researchers suggest that the initial stage of corrosion results in the formation of a compact t-ZrO_2_ oxide film, and as the thickness of the oxide film increases, the compressive stress in the outer oxide film gradually diminishes, leading to a gradual transformation of t-ZrO_2_ into loose m-ZrO_2_. The phase transformation in the oxide film can induce cracks and other defects, thereby accelerating zirconium alloy corrosion [39,40,41]. Based on our findings, it can be concluded that hydrogenation has little effect on the t-ZrO_2_ phase content in the oxide film, and there is no direct correspondence between the t-ZrO_2_ content and the corrosion resistance of Zircaloy-4 in 360 °C/18.6 Mpa/Li + B aqueous solution

The proportions and stresses of m-ZrO_2_ and t-ZrO_2_ in the non-hydrogenated and high-hydrogen samples corroded for 70 days are presented in Table 1. According to the data, the stress in the oxide film of the high-hydrogen sample (420 MPa) is higher than that of the non-hydrogenated sample (380 Mpa). When the stress exceeds a certain threshold, hydrogen diffuses along the stress gradient, leading to accumulation outside the oxide film. This accumulation promotes defect formation such as pores and cracks, facilitating faster diffusion of O^2−^ and OH^−^ towards the O/M interface, ultimately accelerating corrosion [42]. Consequently, it can be concluded that high-hydrogen samples exhibit inferior corrosion performance.

## 4. Conclusions

The corrosion behavior of Zircaloy-4 with varying hydrogen contents in a 360 °C/18.6 MPa/3.5 ppm Li + 1000 ppm B aqueous solution was investigated in this study. The microstructure and phase composition of the oxide film were characterized using SEM, TEM, and Raman spectroscopy. The following conclusions can be drawn:

1. Hydrogenation shortens the corrosion transition time, changes the corrosion kinetics, and increases the corrosion rates of Zircaloy-4. The corrosion resistance of Zircaloy-4 is reduced by 11.5% after the 190-day corrosion when the hydrogen content is increased to 360 ppm.
2. After the 190-day corrosion, the high-hydrogen sample exhibits an increased number and larger size of cracks in the outer region of the oxide film compared to the non-hydrogenated sample. These cracks serve as pathways for O^2−^ diffusion and significantly accelerate the corrosion process of Zircaloy-4.
3. After the 70-day corrosion, the second phases in the oxide films on the non-hydrogenated sample are oxidized. However, in the case of the high-hydrogen sample, the second phases remain stable due to their hydrogen absorption capability. It is possible that an interaction between zirconium hydride and the second phase contributed to accelerated corrosion.
4. After the 70-day corrosion, the high-hydrogen sample exhibits increased stress in the oxide film of approximately 420 MPa, which is larger than that of the non-hydrogenated sample (380 MPa). The larger stress promotes the generation of larger cracks and accelerates the sample corrosion.
5. Pre-hydrogenation has little effect on the t-ZrO_2_ content in the oxide film of the Zircaloy-4, and there is no direct correspondence between the t-ZrO_2_ content and the corrosion resistance of the alloy.

## Figures and Tables

**Table 1:** Proportions and stresses of m-ZrO_2_ and t-ZrO_2_ in oxide films of Zircaloy-4 corroded for 70 d in aqueous solution of 360 °C/18.6 MPa/3.5 ppm Li + 1000 ppm B in non-hydrogenated and high-hydrogen samples.

Specimen	Phase Content (%)		Stress (MPa)
	m-ZrO_2_	t-ZrO_2_	m-ZrO_2_
Without hydrogenation	72.7	27.3	380
High hydrogen	72.5	27.5	420

## Data Availability

The raw data required to reproduce these findings cannot be shared at this time as the data also form part of an ongoing study.
